# Local hopping mobile DNA implicated in pseudogene formation and reductive evolution in an obligate cyanobacteria-plant symbiosis

**DOI:** 10.1186/s12864-015-1386-7

**Published:** 2015-03-17

**Authors:** Theoden Vigil-Stenman, John Larsson, Johan A A Nylander, Birgitta Bergman

**Affiliations:** Department of Ecology, Environment and Plant Sciences, Stockholm University, Science for Life Laboratory, SE-17165, Solna, Sweden; Department of Biology and Environmental Science, Linné University, Science for Life Laboratory, SE-17165, Solna, Sweden; BILS/Swedish Museum of Natural History, Science for Life Laboratory, SE-17165, Solna, Sweden

**Keywords:** Cyanobacteria, Genomic streamlining, Insertion sequences, Local hopping, Nitrogen fixation, Pseudogenes, Symbiosis

## Abstract

**Background:**

Insertion sequences (ISs) are approximately 1 kbp long “jumping” genes found in prokaryotes. ISs encode the protein Transposase, which facilitates the excision and reinsertion of ISs in genomes, making these sequences a type of class I (“cut-and-paste”) Mobile Genetic Elements. ISs are proposed to be involved in the reductive evolution of symbiotic prokaryotes. Our previous sequencing of the genome of the cyanobacterium ‘*Nostoc azollae*’ 0708, living in a tight perpetual symbiotic association with a plant (the water fern *Azolla*), revealed the presence of an eroding genome, with a high number of insertion sequences (ISs) together with an unprecedented large proportion of pseudogenes. To investigate the role of ISs in the reductive evolution of ‘*Nostoc azollae*’ 0708, and potentially in the formation of pseudogenes, a bioinformatic investigation of the IS identities and positions in 47 cyanobacterial genomes was conducted. To widen the scope, the IS contents were analysed qualitatively and quantitatively in 20 other genomes representing both free-living and symbiotic bacteria.

**Results:**

Insertion Sequences were not randomly distributed in the bacterial genomes and were found to transpose short distances from their original location (“local hopping”) and pseudogenes were enriched in the vicinity of IS elements. In general, symbiotic organisms showed higher densities of IS elements and pseudogenes than non-symbiotic bacteria. A total of 1108 distinct repeated sequences over 500 bp were identified in the 67 genomes investigated. In the genome of ‘*Nostoc azollae*’ 0708, IS elements were apparent at 970 locations (14.3%), with 428 being full-length. Morphologically complex cyanobacteria with large genomes showed higher frequencies of IS elements, irrespective of life style.

**Conclusions:**

The apparent co-location of IS elements and pseudogenes found in prokaryotic genomes implies earlier IS transpositions into genes. As transpositions tend to be local rather than genome wide this likely explains the proximity between IS elements and pseudogenes. These findings suggest that ISs facilitate the reductive evolution in for instance in the symbiotic cyanobacterium ‘*Nostoc azollae*’ 0708 and in other obligate prokaryotic symbionts.

**Electronic supplementary material:**

The online version of this article (doi:10.1186/s12864-015-1386-7) contains supplementary material, which is available to authorized users.

## Background

Insertion Sequences (ISs) are defined as genomic sequences of “cut-and-paste” mobile DNA, typically 800-1300 bp in length, which encode the protein Transposase. The Transposase protein consists of a DNA binding domain and a catalytic site with the ability to excise and to reinsert the IS DNA sequence into another position, thus moving an IS from one location in the genome to another [1,2]. In some situations *e.g*. during replication, duplications of IS elements occur, *i.e*. “copy-and-paste” rather than “cut-and-paste”, allowing the IS elements to multiply and proliferate in the genome [3]. As incidents of cut-and-paste events are more difficult to detect than are copy-and-paste incidents, cut-and-paste transpositions are more frequent than implied by the sheer number of IS elements observed in a genome. IS transpositions commonly leave traces in the form of small, difficult to recognize, 2-20 nt direct repeats at the excision site. Insertion sequences accumulate in genomes when the evolutionary pressure is low and when decreases in population size result in genetic drift. They are known to influence the evolution of an organism *e.g*. through gene activation and inactivation, repression, deletions, rearrangements, recombinations, and gene transfers [1]. ISs are considered important for driving reductive evolution in host restricted symbiont microbes [4,5]. For instance, clusters of ISs in genomic “hot spots” have been shown to be correlated with areas of gene loss and genomic recombinations in the gram positive bacterium *Frankia*, strains of which form mutualistic symbioses with plants [6,7]. The same phenomenon has been observed in the intracellular gram-negative bacterium *Shigella flexneri* [8] and in the eukaryotic *Tetraodon nigroviridis* (puffer fish) [9]. In “recent” obligate bacterial symbionts the genome reduction is usually small, while the number of insertion sequences tends to be high. In contrast, in ancient symbiosis the norm is bacterial symbionts with small genomes and few or no ISs [10].

**Figure 1:**
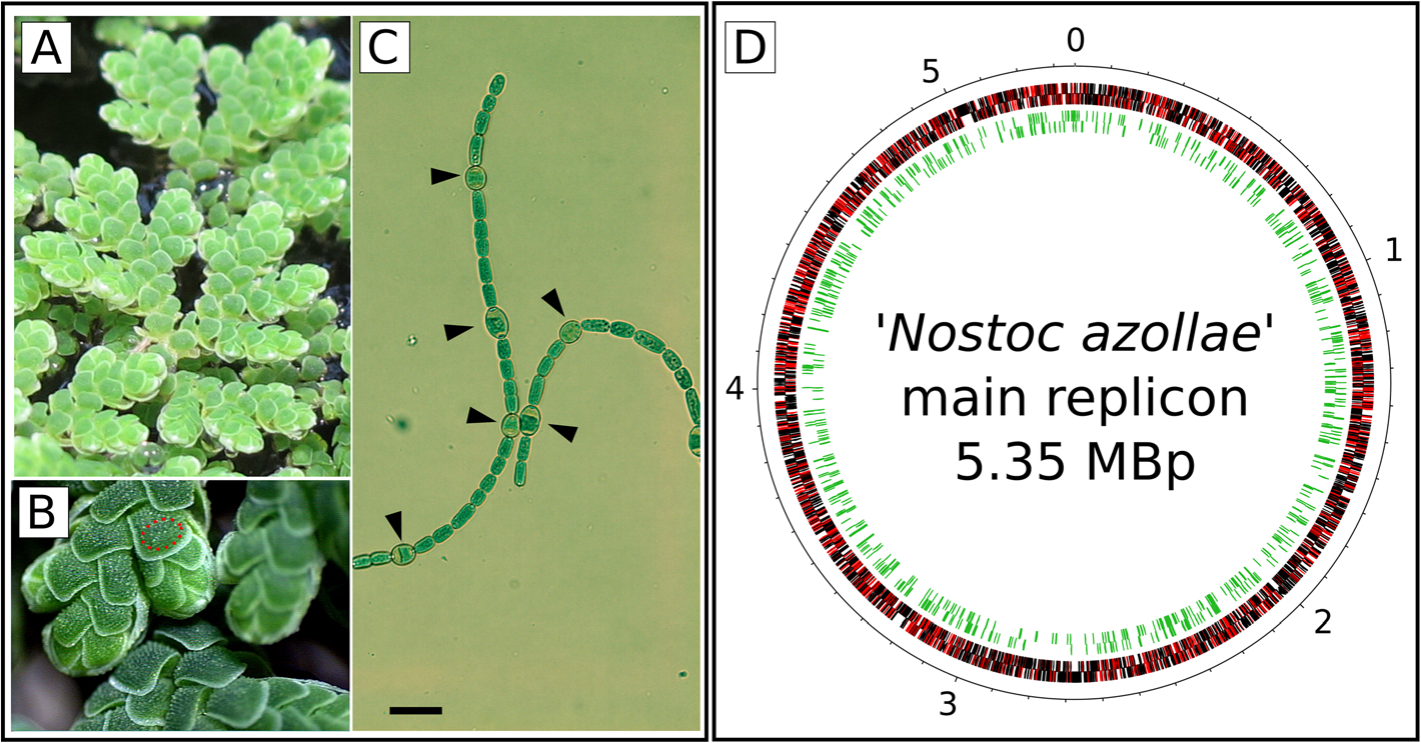
***Azolla filiculoides***, ***the*** **cyanobacterial symbiont** **(NoAz)** **and the NoAz genome. A**. The water-fern *Azolla filiculoides* growing in the greenhouse. **B**. Close-up of *Azolla* leaves. Each leaf contains a specialized cavity (marked with dotted circle) where a colony of ‘*Nostoc azollae*’ 0708 resides. **C**. ‘*Nostoc azollae*’ 0708 filaments. Arrows indicate the differentiated nitrogen-fixing heterocysts. Bar is 10 μm. **D**. Repeat sequences and pseudogenes in the NoAz genome (5.35 Mbps). Red ticks indicate positions of pseudogenes among the black-ticked non-affected functional genes, while green ticks indicate the positions of IS elements within the genome.

In the small floating fern *Azolla* [11] (Figure 1), the symbiotic association with the filamentous cyanobacterium ‘*Nostoc azollae*’ 0708 (from now on abbreviated NoAz) has developed into an extraordinary tight degree [12-14]. During leaf development in *Azolla*, an extracellular cavity is formed in the leaf. These cavities are in nature always occupied by filaments of NoAz. The *Azolla*-NoAz symbiosis is obligate for the bacterial symbiont, as NoAz is unable to grow outside the plant [15] and thus spends its entire life cycle within the plant. Transfer of the cyanobiont (cyanobacterial symbiont) to new plant generations is facilitated by a unique vertical transfer mechanism. Upon formation of the *Azolla* megasporocarp, the female seed-like reproductive structure of *Azolla*, filaments of NoAz are attracted to and enter into a specific ‘chamber’ in this structure, where they reside in a dormant form until germination [13]. When the megasporocarp germinates and develops into new plantlets, the bacterial inoculum differentiates into mobile cells that colonize developing leaf cavities. Inside the cavities, they differentiate again, forming the nitrogen-fixing cell type termed Heterocysts that deliver fixed nitrogen to the plant, thereby satisfying the nitrogen need of the plant.

Due to these intriguing characters we recently sequenced the NoAz genome [14]. Analysis of the genome revealed characteristics of a genome in severe reduction, such as a large number of pseudogenes (1670 out of 5380 genes, or 31% of all genes), a low GC content (38.4%) and genomic streamlining around functions generating critical services for the NoAz-*Azolla* symbiosis, such as those related to nitrogen fixation. We thus proposed that NoAz is forced into an irreversible path of reductive evolution, as also observed in other obligate bacteria-eukaryote symbioses [4], likely to end in a severely reduced genome as seen in ancient symbionts. The cyanobiont will eventually loose autonomy, thereby starting to resemble organelles, such as chloroplasts, that once originated from cyanobacteria [16]. Presently, it is unknown if any NoAz genes has been transferred to the plant nucleus as was the case for chloroplasts. Secreted vesicles containing DNA has been observed in the *Azolla* leaf cavity, [13] and extant chloroplasts may have evolved from a filamentous nitrogen-fixing heterocystous cyanobacterium of the same type as NoAz [17-19], which suggests that DNA transfer is possible.

More than 600 locations with repeated sequences classified as Insertion Sequences were observed in the NoAz genome [14]. Here we define a particular DNA sequence encoding a transposase as an “Insertion Sequence” (IS). An IS is usually present in multiple copies in a genome. We term these copies “IS elements”. Hence, IS elements are the physical copies containing the IS DNA sequence, which in turn may have accumulated mutations leading to changes in sequence and length.

IS element abundance in genomes is proposed to be linked to the high number of pseudogenes observed in some IS rich organisms [20,21]. This pseudogenization may in turn make a symbiont dependent on the host organism and thereby promote the intimacy of the symbiosis.

To further our understanding of events that shaped the eroding genome of NoAz, and the role of the ISs discovered, we examined the identity and distribution of ISs in the NoAz genome in relation to the distribution of pseudogenes. To gain perspective, the IS and pseudogene distribution in the genome of NoAz was compared to 46 other sequenced free-living and symbiotic cyanobacteria. Moreover, nine symbiotic bacteria from a wide range of eukaryotic hosts, were also included in our analysis: *Wolbachia* [22], *Yersinia pestis* [23], *Frankia* [24], *Candidatus Amoebophilus asiaticus* [21], *Bordetella pertussis* [25], *Orientia tsutsugamushi* [20], *Shigella flexneri* [8], *Mycoplasma mycoides* [26], and Onion yellows phytoplasma [27] (Table 1, Additional file 1).

**Table 1:** **Organisms included in the study**

**Organism**	**Abbreviation**	**Genome size** **(Mbp)** ^**a**^	**GC content**	**Habitat**	**Morphology**	**Symbiotic state**	**Total repeathits**	**Total IS hits**	**Annotated pseudo-** **genes**	**Repeats/** **Mbp**	**Pseudo-** **genes/** **Mbp**
Cyanobacteria											
*Acaryochloris marina* MBIC 11017	Acam	8.36	0.47	Marine	unicellular	Free-living	588	553	32	70	4
*Anabaena variabilis* ATCC 29413	Anav	7.11	0.41	Terrestrial	filamentous, heterocystous	Free-living	329	313	40	46	6
*Arthrospira maxima* CS-328	Artm	6.00	0.45	High-salt lakes	filamentous, non-heterocystous	Free-living	1208	906	0	201	0
*Arthrospira platensis str. Paraca*	Artp	5.00	0.44	High-salt lakes	filamentous, non-heterocystous	Free-living	206	141	0	41	0
*Crocosphaera watsonii* WH 8501	Crow	6.24	0.37	Marine	unicellular	Free-living	1611	1494	0	258	0
cyanobacterium UCYN-A	Ucyn	1.44	0.31	Marine, Host: prymnesiophytes	unicellular	obligate unclassified symbiont	1	1	0	1	0
*Cyanothece* sp. PCC 8802	Cya8802	4.80	0.40	Marine	unicellular	Free-living	203	176	204	42	43
*Cyanothece*	Cya	6.09^5^	0.41	Marine	unicellular	Free-living	246	205	167	40	27
*Cylindrospermopsis raciborskii* CS-505	Cylr	3.88	0.40	Freshwater lakes	filamentous, heterocystous	Free-living	517	303	1	133	0
*Gloeobacter violaceus* PCC 7421	Glov	4.66	0.62	Terrestrial	unicellular	Free-living	143	82	0	31	0
*Lyngbya* sp. PCC 8106	Lyns	7.04	0.41	Marine, Freshwater	filamentous, non-heteroc	Free-living	443	339	0	63	0
*Microcystis aeruginosa* NIES-843	Mica	5.84	0.42	Freshwater lake	unicellular	Free-living	1664	1563	0	285	0
Nodularia spumigena CCY9414	Nods	5.32	0.41	Brackish water	filamentous, heterocystous	Free-living	352	294	0	66	0
‘*Nostoc azollae* 0708’	NoAz	5.49	0.38	Host: Azolla ferns	filamentous, heterocystous	obligate extracellular symbiont	970	907	1670	177	304
*Nostoc punctiforme* PCC 73102	Nosp	9.06	0.41	Terrestrial	filamentous, heterocystous	facultative intracellular	399	366	371	44	41
*Nostoc* sp. PCC 7120	Noss	7.21	0.41	Terrestrial	filamentous, heterocystous	Free-living	311	278	0	43	0
*Prochlorococcus marinus* strains	Prom	1.86^12^	0.36	Marine	unicellular	Free-living	18	12	15	10	8
*Raphidiopsis brookii* D9	Rapb	3.19	0.40	Freshwater lake	filamentous,non-heterocystous	Free-living	109	80	0	34	0
*Synechococcus elongatus* species	Syne	2.7^2^	0.55	Freshwater lake	unicellular	Free-living	8.00	7.00	2	3	1
*Synechococcus* JA species	SynJA	2.99^2^	0.59	Hot spring	unicellular	Free-living	331	330	51	111	17
*Synechococcus* species	Syn	2.5^7^	0.56	Marine	unicellular	Free-living	6	5	10	2	4
*Synechocystis* sp. PCC 6803	Scys6803	3.95	0.47	Freshwater lake	unicellular	Free-living	161	151	0	41	0
*Thermosynechococcus elongatus*BP-1	Thee	2.59	0.54	Hot spring	unicellular	Free-living	128	99	0	49	0
*Trichodesmium erythraeum* IMS101	Trie	7.75	0.34	Marine	filamentous,non-heterocystous	Free-living	1311	1144	625	169	81
Non cyanobacterial symbionts											
*Bordetella pertussis* Tohama I	Borp	4.09	0.68	Host: Human	unicellular	obligate intracellular	388	380	358	95	88
*Candidatus amoebophilus asiaticus* 5a2	Cana	1.88	0.35	Host: Acanthamoeba	unicellular	obligate intracellular	315	273	222	168	118
*Frankia*	Fra	7.00^5^	0.71	Host: Plants	filamentous	facultative intracellular	139	130	53	20	8
Onion yellows phytoplasma OY-M	PhyOY	0.85	0.28	Host: Plants	unicellular	obligate intracellular	113	29	0	133	0
*Orientia tsutsugamushi* str. Boryong	Orit	2.13	0.31	Host: Human	unicellular	obligate intracellular	1122	703	997	527	468
*Shigella flexneri* 2a str. 2457 T	Shif	4.60	0.51	Host: Human	unicellular	facultative intracellular	466	450	378	101	82
*Mycoplasma mycoides* subspecies	Myc	1.18^2^	0.24	Host: Animals	unicellular	obligate intracellular	77	75	1	65	1
*Yersinia pestis* strains	Yer	4.8^2^	0.48	Host: Animals	unicellular	facultative intracellular	306	294	155	63	32
*Wolbachia* subspecies	Wol	1.38^6^	0.35	Host: insects and nematodes	unicellular	obligate intracellular	361	161	67	260	48

^a^Several similar bacteria are included in entries with superscripts. Genome sizes are averages, with the number of species indicated by the superscript.

Table of genomes included in the study. Very similar organisms, e.g. several genomes of Cyanothece, have been included as a single row, and the numbers therein represents averages of the data found. The numbers of organisms included in such groups are indicated with a superscript in the Size column of the table. Total repeat hits: The number of hits received to repeated sequences of any kind. Repeated genes with known functions that are not mobile DNA are not included in this count. Total IS hits: number of hits received to repeated sequences that are Insertion Sequences. Annotated pseudogenes: number of pseudogenes in the genome, according to the integrated microbial genomes and metagenomes database (IMG Data Management and Analysis Systems). The following two columns show number of repeated sequences and pseudogenes per Mbps of genome.

The results show that NoAz has a high portion of its genome occupied by IS elements: 14.3%, which is just below the proportion in *O. tsutsugamushi* (14.7%), but higher than in any of the other symbiotic organism investigated. Still, some non-symbiotic cyanobacteria had higher or equal IS contents to that of NoAz, e.g. the unicellular *Crocosphaera watsonii* (23.1%) and *Microcystis aeruginosa* (14.3%), pointing to multiple roles and consequences of IS elements in free-living and symbiotic bacteria. The observed co-location of IS elements and pseudogenes, as well as the tendency for some IS elements to perform “local hopping” suggests a model for the role of ISs in reductive evolution which is discussed.

## Results

**Figure 2:**
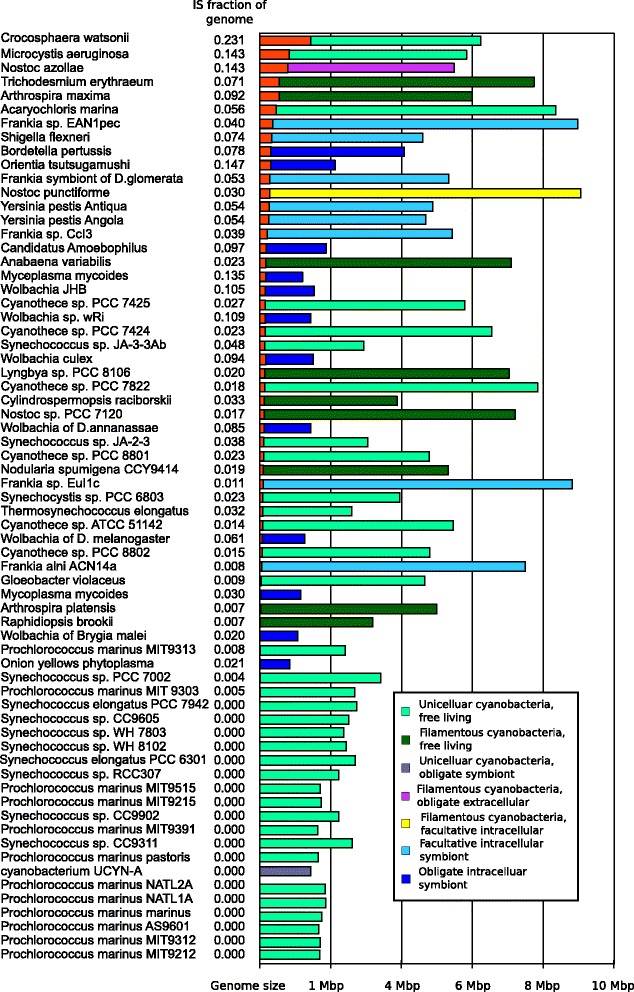
**Genome size and repeat density in investigated genomes.** Genomes are ordered by amount of IS base pairs. IS content is displayed in red, while the rest of the bars indicate genome size and symbiotic state.

Using the repeat identification software, BLAST and in-house python scripts, a total of 1108 different repeated sequences of a length above 500 bp (Additional file 2 and Additional 3) were identified in the totally 67 bacterial genomes investigated (Figure 2). These 1108 repeated sequences comprised 19841 copies or fragments in the genomes. Of the 1108 repeated sequences, about half or 578 sequences (comprising 4489 copies) belonged to previously annotated ISs identified and catalogued on the ISfinder website [28]. Of the remaining 539 sequences, the majority or 419 sequences (11794 copies) showed similarities to known IS sequences. Of the total number of repeats, fifty sequences (926 copies) appeared to be of phage origin. These were found almost exclusively in *Wolbachia*, *O. tsutsugamushi* and ‘Onion yellows phytoplasma’, with phage contents of 2-6% of the genomes. ‘Onion yellows phytoplasma’ with the smallest genome, 0.85 Mbp, still diverted 7.7% of its genome to be occupied by IS elements and phages, and was the only organism with more phage remains than IS elements.

**Figure 3:**
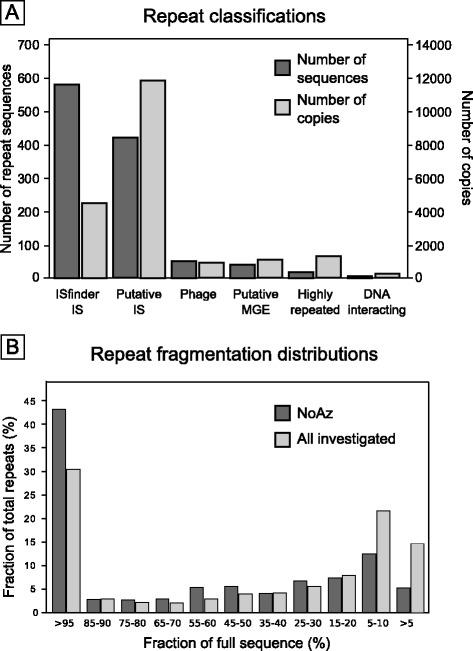
**Repeat classifications and fragmentation of repeat sequences in bacteria.** Panel **A**: Number of repeat sequences and copies found in the 67 genomes of the investigated bacteria. Notably, the majority of the discovered repeats are IS elements, while phages and unidentified repeats were low. Number of repeat sequences refers to the number of different repeat sequences found for each class of repeats. Number of copies found refers to the total number of repeat copies found for each class of repeat. ISfinder IS: Repeats listed in the ISfinder database. Putative IS: Repeats with blast similarities to Insertion Sequences. Phages: Repeats with blast similarities to phage genes. Putative MGE: Repeats with no decisive data on kind of replication mechanism, but which are nevertheless suspected to be self-replicating and/or mobile when their similarities to other kinds of Mobile Genetic Elements are considered. Highly repeated: Repeats with low similarities to known proteins, but which appear in high copy numbers in the investigated organisms. DNA interacting: Repeats which are similar to proteins interacting with DNA (*e.g*. nucleases, helicases), making mobility and/or self-replication feasible. Panel **B**: Repeat fragment size distribution. Distribution of fragment lengths in NoAz compared to the average distribution in all other 66 bacteria investigated. X-axis: Fragment size in per cent of full size. Y-axis: Frequency of fragment size, expressed as percentage of total number of copies.

Sixty-one sequences (2632 copies) were putatively self-replicating because of their high copy numbers and/or similarities to genes with functions related to gene replication or transposition, *e.g*. endonucleases, RNA directed DNA polymerases and helicases. Each group of repeats contributed to the total amount as illustrated in Figure 3A. Repeats with IS characteristics were predominant both in diversity (number of different nucleotide sequences) and abundance (copy number).

The repeats showed considerable fragmentation: of the total number of IS elements in the 67 genomes examined 31% showed a length over 95% of its reference sequence, and 37% of the repeats were less than 15% of the sequence length (Figure 3B).

### Insertion sequences in NoAz

In NoAz, a total of 907 IS elements occupied 14.3% of the total genome. These IS elements were in various states of preservation, but the majority had a length ≥ 95% of its corresponding IS sequence. At the same time, a total of 1670 genes were annotated as pseudogenes in the NoAz genome, almost double the amount annotated in any other bacterium examined here.

The size of the NoAz genome with all IS elements removed is 4.67 Mbps, which is considerably larger than the IS-free genome size of its two closest free-living cyanobacterial relatives *Raphidiopsis brookii* (3.16 Mbps) and *Cylindrospermopsis raciborskii* (3.69 Mbps). However, if the 1670 pseudogenes in NoAz (covering 1.5 Mbps), are also removed the sizes start to match.

**Figure 4:**
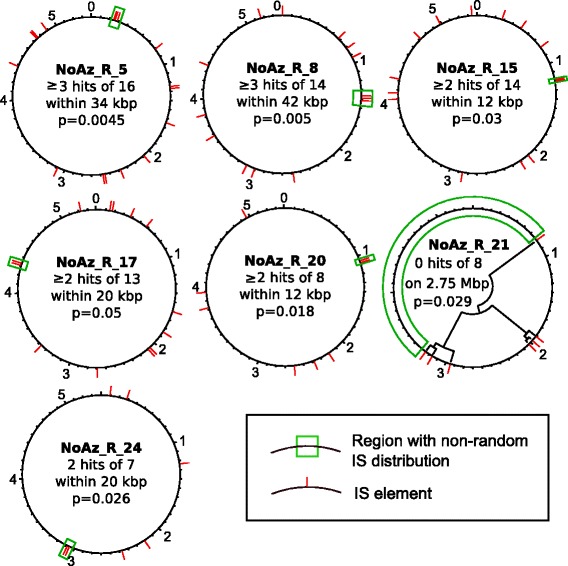
**Genomic areas with non**-**random IS density in the cyanobacterial symbiont NoAz.** Locations of ISs >70% target length for selected repeats in NoAz. Red ticks indicate IS locations. The area of interest is marked with green borders. In the middle of each circle is listed the probability for the IS elements of interest to come in close proximity by chance alone, and the data used to compute this probability. For NoAz_R_21, a circular tree based on uncorrected p-distance (see Methods) has been superimposed on the genome, showing that elements that are similar in nucleotide sequence are located close to each other. NoAz_R_21 elements were chosen in this example because of the simplicity of display, other IS elements make up similar albeit more tangled trees. Numbers at tick marks show genomic position in Mbps.

**Figure 5:**
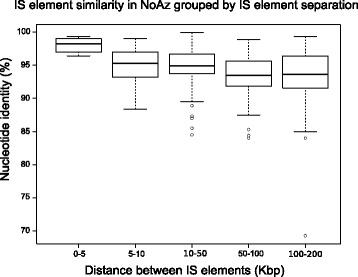
**Box plot of uncorrected p**-**distance by separation distance in NoAz.** Box plot graph comparing uncorrected p-distance between pairs of IS elements at different distances (in bp) from each other. Boxes show inner quartiles.

Copies of the same IS tended to cluster together in the genome of NoAz, or to be absent from a large region (Figure 4). IS elements in close proximity to each other were also more similar in nucleotide identity than were IS elements distant from each other (Figure 5, Additional file 4). In some cases, the most proximal ISs on the chromosome were also closest in terms of sequence identity (uncorrected p-distance) (Figure 4, NoAz_R_21).

**Figure 6:**
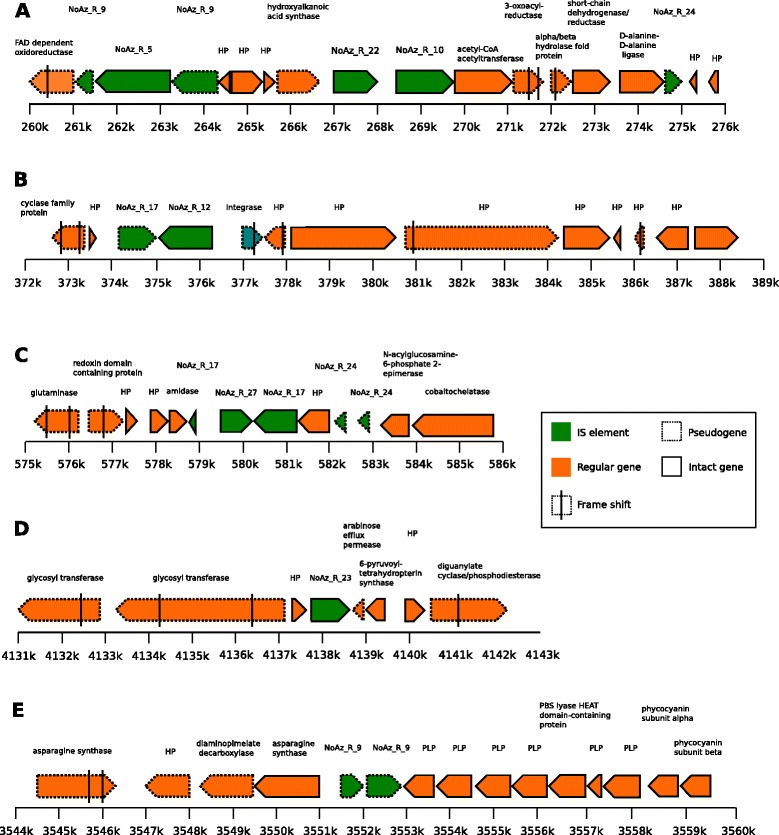
**IS elements in proximity to pseudogenes in NoAz.** IS elements in proximity to pseudogenes in the NoAz genome. The selected examples show both IS elements and regular genes in close proximity to IS elements being converted into pseudogenes. Green arrows represent IS elements and orange arrows regular non-affected genes. Genes annotated as pseudogenes are illustrated by dashed borders. Frame shifts, where known, are indicated by black vertical bars. HP = Hypothetical Protein. PLP = Phycobilisome Linker Polypeptide. Ruler indicates position on the main chromosome. **A**: The IS element Noaz_R_5 inserted into Noaz_R_9, generating flanking pseudogenes. Further down are two full-length NoAz IS elements, NoAz_R_22 and NoAz_R_10, with flanking pseudogenes. **B**: NoAz_R_12 surrounded by pseudogenes with frame shifts. Most genes are hypothetical or other insertion sequences. **C**: The IS element NoAz_R_27 may have fragmented surrounding genes and pseudogenized a glutaminase gene. **D**: NoAz_R_23 surrounded by pseudogenes. **E**: NoAz_R_9, itself probably pseudogenized, may have generated the pseudogenes on the left. The psychobilisome linker polypeptide proteins to the right of NoAz_R_9 are probably critical to NoAz fulfilling its symbiotic role, and therefore intact.

As seen in the graphical representations of IS element and pseudogene positions in the NoAz genome these are frequently located in proximity to each other (Figure 1D). This is to some degree due to the tendency for IS elements, many of which are pseudogenes, to cluster (together) in the genome. However, it is apparent that both other IS elements and regular genes are pseudogenized in the vicinity of IS elements (Figure 6A-D). Exceptions are genes that are presumably critical to symbiotic functions, which are maintained intact despite their proximity to presumably active IS elements (Figure 6E).

### Insertion sequences in other cyanobacteria

**Figure 7:**
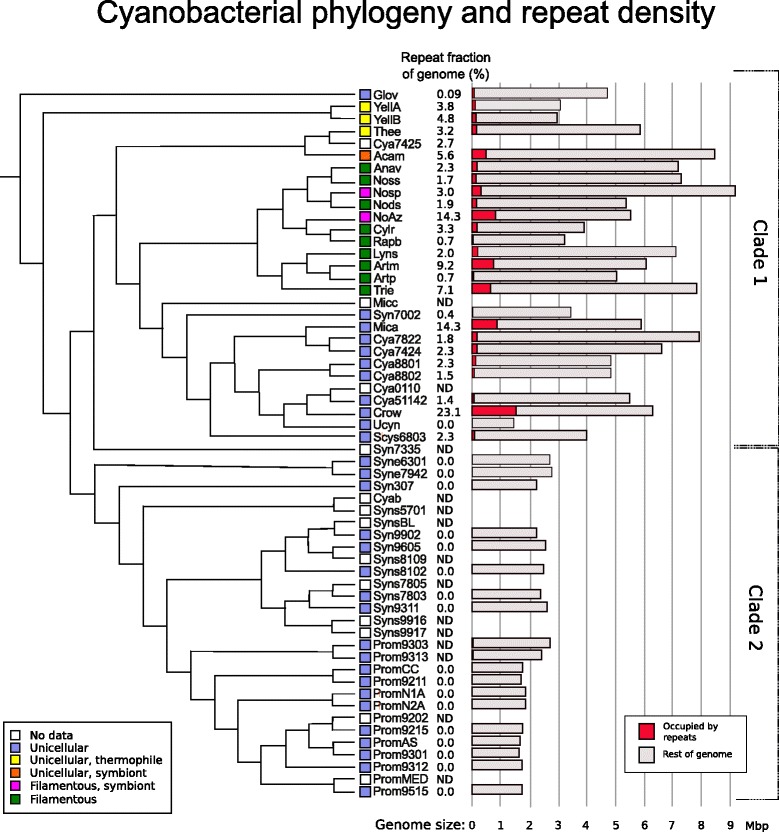
**Phylogeny and repeat density in genomes of cyanobacteria.** Phylogenetic tree depicting the two major cyanobacterial clades, with Clade representing cyanobacteria with primarily larger genomes and Clade 2 unicellular cyanobacteria with smaller genomes (after [12]. Morphology and symbiotic state are indicated by coloured squares. Open squares and “ND” in “% occupied” indicate that this bacterium was not included in the study. Genome size (light grey bars) and portion occupied by repeats (red) are displayed on the right.

**Figure 8:**
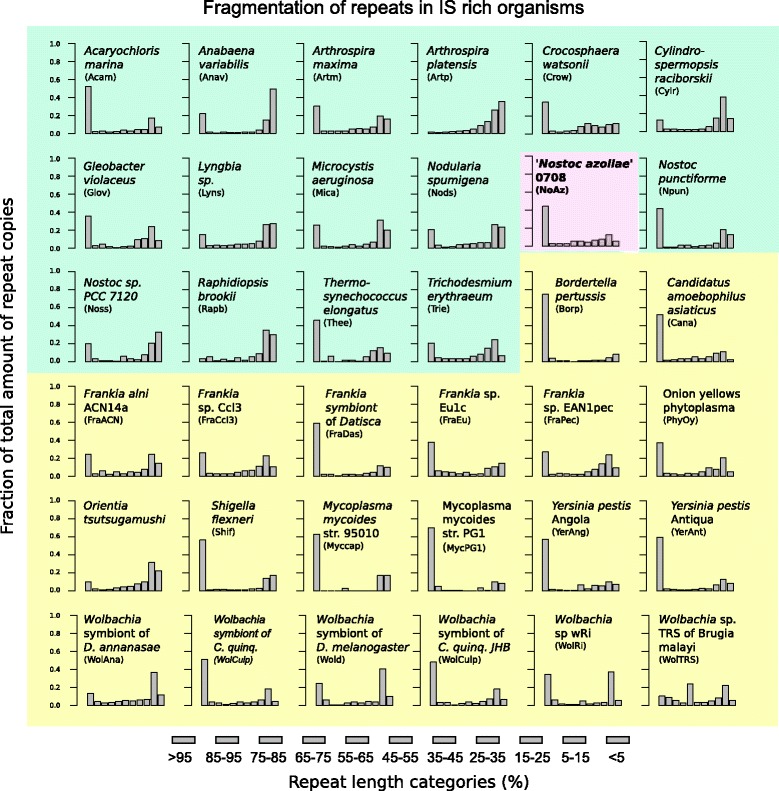
**Fragmentations of repeats in IS rich bacteria.** Bars represent the fraction of repeats copies found for a certain size category. Light green graphs represent 16 cyanobacteria, with NoAz given in pink and light yellow bars represent the other 20 investigated bacteria. The total of the bar heights for each graph is 1. X-axis: Fragment size in per cent of full size. Y-axis: proportion of total repeat copies that belong to a certain size category.

The high IS concentration in NoAz (14.3%) is unique among the seven multicellular and highly differentiated heterocyst-forming cyanobacteria investigated (Figure 7). In fact, NoAz contains more than three times more IS elements than these close relatives, whether symbiotic or free-living. In contrast, some more distantly related and less differentiated (non-heterocystous) relatives showed IS contents equal to or exceeding that of NoAz. The IS content of the single-celled *Microcystis aeruginosa and Crocosphaera watsonii* were 14.3% and 23.1%, respectively. *C. watsonii* is an abundant oceanic nitrogen-fixer [29], while *M. aeruginosa* is a widespread toxin producing freshwater species unable to fix nitrogen. Moreover, in some cases, closely related cyanobacteria show extreme differences in IS content. This is the case within the genus *Arthrospira*, with the halophilic *A. maxima* having an IS density of 9.2%, *versus* the freshwater *Arthrospira platensis* with merely a 0.7% IS density and also in *C. watsonii* (23.1%) versus the recently discovered nitrogen-fixing but symbiotic cyanobacterium UCYN-A [30] (lacking ISs). This is also the case for NoAz and its two close relatives *C. raciborskii* and *R. brookii*, *with the former* showing 133 IS hits per Mbp, comparable to 177 IS hits per Mbp in NoAz. On the other hand, the *C. raciborskii* fragments are truncated to a larger extent (Figure 8), making the fraction of the IS occupied genome significantly smaller (3.3%) than in NoAz. While the two *Arthrospira* species occupy widely different habitats (saline/freshwater) the differences in IS abundance in the two fresh-water species *C. raciborskii* and *R. brookii* cannot be attributed to different life styles.

Notably, the IS abundance in cyanobacteria does not correlate with either symbiotic state, morphology or phylogeny, ISs rather appear to be particularly prevalent in the clade comprising complex cyanobacteria with large genomes ((Figure 7, Clade 1 [12]). Clade 2, which consists exclusively of single-celled species with small cell size and streamlined genomes, such as marine *Prochlorococcus* and *Synechococcus*, are almost completely devoid of IS elements or DNA repeats of any kind (Figure 7).

### Insertion sequences in non-cyanobacterial species

Symbiotic bacteria outside the cyanobacterial phylum showed a wide range of IS densities, 0.8-14.7%, with a median of 5.7% (Figure 2). As in cyanobacteria, genomes with a high IS frequency also tended to have a large proportion of full length IS elements. Most symbiotic bacteria also contained a high proportion of pseudogenes in their genomes, 6-12%, *i.e*. a considerably higher frequency than in cyanobacteria (0-5%, except for NoAz and *T. erythraeum*). Bacteria belonging to the gram positive genus *Frankia* in general contained low densities of IS elements, 1-2%, and few pseudogenes, except for the symbiont of *Datisca* with 5% IS elements and 7% pseudogenes.

### Repeat fragmentation

Among the bacteria with a relatively high repeat density (>3%), full-length IS elements were in majority (Figure 3, Figure 8). Bacteria with a low density of IS elements showed a larger proportion of fragmented IS elements, with lengths <50% of the reference sequence. The occurrence of intact IS elements in IS rich genomes is particularly obvious in NoAz, where over 40% of the IS elements being full-length. Yet another example is in the difference between *A. Maxima*, with 9.2% IS elements, out of which 30% is full-length and its closest relative *A. platensis* with only 0.7% IS elements, and 1.5% full-length IS elements.

### New IS elements

Full length-copies of 210 previously annotated ISs were identified in our investigation. For example, IS895 [31] and IS100kyp from *Y. pestis*, IS892 [32] from the free-living cyanobacterium *Nostoc* PCC 7120 and ISNpu1 – ISNpu13 found in the symbiotically competent cyanobacterium *N. punctiforme* [28]. Another 362 previously annotated ISs were found as fragments, being <95% of their full lengths.

In addition, a number of previously unannotated (<95% nucleotide similarity to annotated ISs) repeats were identified in the 67 genomes (Additional file 3). For instance, 24 previously unannotated putative ISs were found in NoAz, perhaps not surprising considering its high frequency of IS elements. The found sequences in NoAz varied widely as for previously identified ISs. For example, NoAz_R_22 is 86% identical to the nucleotide sequence of the previously annotated ISAva8, while NoAz_R_21 is a “hypothetical protein” where only a stretch of 100 amino acids are similar (77%) to known ISs. The IS repeats in NoAz were most similar to previously identified ISs from close relatives: *N. punctiforme* (Noaz_R_10, NoAz_R_19), *Anabaena variabilis* (NoAz_R_2, NoAz_R_22, NoAz_R_24), or from other IS-rich cyanobacteria: *Acaryochloris marina* (NoAz_R_17, NoAz_R_13, NoAz_R_14) and *C. watsonii* (NoAz_R_11, NoAz_R_18).

Unannotated putative ISs were also found previously annotated genomes. For instance, the sequence Npun_R_21 found in *N. punctiforme* was present in 28 full-length copies, and WolAna_R_20, a 538 long hypothetical protein, was found in 27 full-length copies in the *Wolbachia* genomes. The unicellular free-living cyanobacterium *M. aeruginosa* contained the highest frequency of unannotated ISs, with at least thirty sequences clearly of IS character. At least one IS in NoAz, NoAz_R_12, appeared spread among close relatives. This IS701 family IS, with 28 full-length repeats in NoAz, has well-preserved (blastn scores > 1300) copies in cyanobacteria such as *N. punctiforme*, *Anabaena cylindrica*, *Calothrix* sp. PCC 7507 and *Calothrix* sp. PCC 6303.

### Insertion sequences display ‘local hopping’

It has previously been reported that some ISs in *Escherichia coli* transpose short distances rather than moving randomly across the genome [33]. This appears to hold true for some of the ISs in NoAz as well (Figures 4 and 5). To determine whether these patterns exist in other bacteria, linear regression tests were performed on 100 of the most frequent ISs in the investigation. Uncorrected p-distances between two IS elements in the genomes were tested as a variable dependent on the length of nucleotides that separated them (see Methods). Seventeen of the 100 ISs investigated showed significant positive relationships between separation of the genome and p-distance. These 17 ISs tended to be less similar to each other the further they were separated. On average, the uncorrected p-distance between two IS elements decreased with 0.1-1 percentage unit per Mbp as their distance from each other increased. This means that 1-10 base pairs have mutated for every Mbp of separation (disregarding reversions). Tests searching for a negative correlation were not found. A tendency to perform “local hopping” is particularly apparent in NoAz. Ten IS species in NoAz, out of twenty investigated, showed a significant negative correlation between alignment similarity and genomic separation. When the 49 757 pairs of IS elements from the 100 ISs were tested together, an average change in uncorrected p-distance of -0.48 percentage units per Mbp of distance was found using linear regression, with a p-value of 2.35e-25.

### Pseudogenes in proximity to insertion sequences

**Figure 9:**
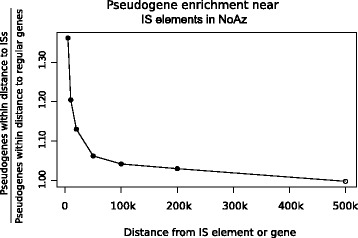
**Pseudogene enrichment with increasing distance from IS elements in the symbiotic cyanobacterium NoAz.** X-axis: Distance from gene or IS elements within which all pseudogenes are counted. Y-axis: Pseudogene enrichment, i.e. (average number of pseudogenes within distance to IS elements)/(average number of pseudogenes within distance to regular genes). Filled circles indicate that the difference in pseudogene enrichment is statistically significant (p < 0.05), empty circles indicate that the difference is not significant.

**Figure 10:**
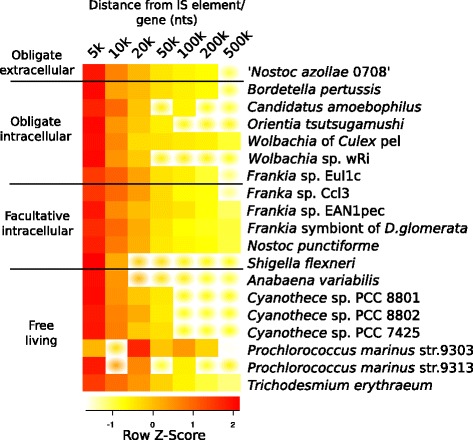
**Pseudogene enrichment with increasing distance from IS elements in genomes of bacteria with different life styles.** Heat map depicting pseudogene enrichment at seven distances from a gene or IS element in symbiotic and free-living cyanobacteria and in obligate or facultative intracellular bacteria. Pseudogene enrichment is computed as (avg. number of pseudogenes within [distance] of IS element)/(avg. number of pseudogenes within [distance] of annotated gene). Increasing red colours indicate a higher quotient. Blurred squared indicate that the difference in pseudogene enrichment between IS element and annotated genes is not statistically significant (p ≥ 0.05). Organisms with less than two significant points were omitted, as were those with no annotated pseudogenes.

A graphical representations of the genome in NoAz illustrating the comparatively high numbers of IS elements and pseudogenes, revealed a pattern with pseudogenes appearing more frequently in the vicinity of IS elements. To test if this applied to other organisms, the average number of pseudogenes near IS elements was computed in the 67 genomes and compared to the average number of pseudogenes near regular genes. The test showed a significant positive correlation increase (Wilcoxon rank sum test) in the average number of pseudogenes close to IS elements, in comparison to the average number of pseudogenes in the vicinity of regular genes. In NoAz there appeared to be, on average, >30% more pseudogenes within 5 kbp of an IS element. This enrichment dropped to below 5% at a 200 kbp distance and became undetectable at 500 kbp distance (Figure 9). Similar patterns were apparent in the majority of the investigated organisms holding a sufficient number of IS elements and pseudogenes make statistical relevant (p < 0.05) differences (Wilcoxon test), such as *N. punctiforme*, all *Frankia* species except *Frankia* ACN, and most *Cyanothece. Orientia tsutsugamushi* showed significant pseudogene enrichment in the 20 kbp closest to IS elements, while the cyanobacterium *A. variabilis and the bacteria B. pertussis*, *C. amoebophilus*, *M. mycoides*, *S. flexneri* and the *Wolbachia* symbionts showed the same trend but with lower significance (Figure 10).

The average enrichment of pseudogenes ranged from 20% to 200% within the first 5 kbp distance from IS elements and dropped to a fourth of this at 200 kbp distance. At 500 kbp distance from IS elements, most of the investigated organisms showed no statistically significant pseudogene enrichment Additional file 5).

## Discussion

When a bacterium is sheltered by a “host” organism, the host typically provide functions originally handled by the microorganism, making some genes in the symbiont superfluous. Such redundant genes may be colonized by mobile DNA elements and interrupted. The damage to the microorganism fitness is then minimal as it lives in a relaxed habitat of a eukaryotic host, plant or animal. The presence of superfluous genes may thus lead to an increase in the number of IS elements in the genome, as there may be no negative consequence and as these genes offer sites for safe insertion of IS elements. However, a large number of active IS elements in the symbiont means a greater risk for critical genes being negatively affected or even destroyed by transposition events, hampering the behaviour of the microorganisms and leading to slower growth of the symbiont. This may, up to a point, be beneficial to the host, as it may benefit from a fixed small bacterial population rather than a freely expanding one.

Decreases in IS abundance is facilitated in two ways, via i) inactivation of insertion elements by natural mutations and by pseudogenization, sometimes brought about by other IS elements, and via ii) deletions of large regions of the genome through recombination events. Bacteria seem to have a bias towards genome reduction [10], and the presence of many repeated elements may itself help to reduce genome sizes by providing repeated sequences able to participate in homologous recombinations.

Other investigators have focused on genetic exchange as a major factor for IS abundance, and argue that facultative symbionts should have a high IS abundance because of genetic inflow from their symbiotic partners, while obligate symbionts should have a low frequency of ISs due to their isolation. This trend was proven in an investigation of 384 bacterial genomes in [34]. However, the organisms investigated here do not show the same trend, instead obligate symbionts harboured more IS elements than facultative symbionts. This difference may be due to a smaller and more biased set of genomes analysed here, but it could also be a result of the relaxed selection pressure that are imposed on early obligate endosymbionts before their genomes become smaller and more vulnerable to change. Since NoAz is an obligate inhabitant of a semi-open cavity shared with other microorganisms [35], the IS content is likely influenced both by a relaxed selection pressure and genetic exchange.

The genome of the symbiont of *Azolla* suggests that it may be in the middle of an evolutionary scenario of IS-mediated reductive evolution. This is concluded from the fact that it holds a high portion of IS elements and pseudogenes, while for instance its nitrogen-fixing function (and all *nif* genes) are still preserved [14]. As many of the ISs in the NoAz genome are present in high copy numbers (10-80 copies), and the majority of IS elements found are full length, NoAz may have an active contingent of IS elements that continue to shape its genome. An active IS population is however not limited to NoAz: several other of the investigated bacteria, both symbiotic and free-living, show a considerable level of IS elements, and also tend to have a high proportion of full-length copies. In free-living organisms, functions lost due to IS insertions cannot be substituted by a symbiotic host, and hence, other mechanisms of surviving contingents of active ISs in the genome must be in operation. One mechanism may be to hold extended ‘safe’ genomic portions unaffected by IS element insertions *e.g*. non-coding regions, or to allow some degree of IS insertions, *e.g*. in duplicated genes of larger genomes. Many IS-rich cyanobacteria do show a large number of paralogous genes [12], and most of the other IS rich relatives analysed here have genome sizes above average even for Clade I cyanobacteria (Figure 7). Large and flexible genomes may also be prone to take up ‘compensatory’ genomic material from the environment via horizontal gene transfer which may outweigh the loss of productivity caused by lethal IS insertions. Compared to NoAz and non-cyanobacterial symbionts, the free-living cyanobacteria investigated here show a considerably lower number of pseudogenes, with the exception of the filamentous cyanobacterium *T. erythraeum*. Although differences in pseudogene annotation cannot be ruled out, such data suggest a higher sensitivity to pseudogenization in free-living organisms and that individuals with a high load of pseudogenes simply do not survive.

The tendency for pseudogenes to appear in proximity to IS elements highlights the dynamic nature of “cut-and-paste” mobile genetic elements. Genomes with high levels of intact IS elements represent snapshots of IS positions at this moment in time, and these IS elements may have transposed in recent evolutionary time. We propose here that the pseudogenes observed close to IS elements in NoAz, as well as in the other organisms, may be genomic scars from recently transposed IS elements, currently located some distance away. This behaviour of ‘local hopping’ and pseudogenization would favour reductive evolution of the genome, as regions containing superfluous genes will accumulate IS elements. This in turn will i) increase the chance of inactivation of genes in the same genomic region and ii) increase the number of repeated sequences in the region, thus increasing deletion instances of this area. Proximity between IS elements and pseudogenes also implies a higher risk for pseudogenes than for functional genes to be included in a deleted region.

Since it is not known how much of the NoAz genome that is still superfluous to the contemporary NoAz-*Azolla* symbiosis, it is hard to determine whether the IS concentration in the genome of NoAz is still increasing, or whether it has reached a stable IS population fit for this particular plant symbiosis at this stage. Along with the deletion of pseudogenes and superfluous regions, the number of safe spaces for IS insertion will decrease, which in turn will increase the risk for hosting active IS elements and favouring their inactivation and ultimate deletion. Our estimates predict that the genome of NoAz will decrease a conservative estimate of at least 1 Mbp, once all IS elements and pseudogenes have been removed, leaving a genome size of approximately 4.5 Mbps.

## Conclusions

Our results show that the 24 verified or putative species of IS elements in the symbiotic cyanobacterium NoAz cover 14.3% of its genome, which is comparable to the IS density in the obligately symbiotic pathogens *O. tsutsugamushi* (previously *Rickettsia tsutsugamushi*) and *Mycoplasma mycoides*, but notably also to the IS frequency in the free-living cyanobacteria *M. aeruginosa* and *C. watsonii*.

The prevalence of pseudogenes is linked to the prevalence of IS elements, and we suggest that the IS-proximal pseudogenes may be the result of “local hopping” of the IS elements. We propose that this process facilitates the reductive evolution discovered recently [14] not only in NoAz/*Azolla* but also in other well-studied symbiotic bacteria such as *Wolbachia* and *O. tsutsugamushi*.

## Methods

### Finding and identifying repeats

To identify ISs in the investigated prokaryotic organisms, a database of potential ISs was assembled from two sources: Firstly, the program RepeatScout [36] was used to identify all repeated nucleotide sequences with >500 nt length in the investigated organisms. Repeats associated to non-mobile repeated genes, e.g. rRNA, photosynthesis genes and other regular genes present in multiple copies in the genome, were removed.

Secondly, all IS annotated nucleotide sequences, in total 3377 in July 2012, were downloaded from the ISfinder site [28], a web repository of known ISs. The repeats found by RepeatScout were named with an abbreviation of the name of the originating organism, the letter ‘R’ and a number, e.*g*. ‘Npun_R_21’ is a repeat from the organism *N. punctiforme*, the twenty-first found by RepeatScout. The ISs from ISfinder retained their original names, all starting with ‘IS’. ISfinder and RepeatScout sequences were collected into a single Fasta file. Redundancies, generated when ISfinder sequences were also detected by RepeatScout, were removed, with the ISfinder name taking precedence.

Each of the collected nucleotide sequences was used as query in a Blastn search against each of the genomes, one at a time. All areas of the genome that received hits with a Blastn expect value < 1e-6 were collected. Often several queries scored hits on the same region of a genome, together making up a “footprint” in the genome.

In the next step, each footprint was analysed to determine what repeats it consisted of. This was performed by using the footprints as queries against the database of repeats. The repeat sequence that received the highest score with the footprint as query was chosen as the most likely IS to occupy the footprint. In some cases, the best scoring repeat didn’t cover the entire footprint. The search was then repeated with the remaining footprint as query. The process was repeated until all parts of the footprints had been identified.

Next, the identity of the repeated sequences was analysed by using the repeats as queries in a Blastx search against NCBI’s nr database. Repeats that showed Blastx similarity to transposases with an expect value of E < 1e-5 were considered putative ISs. Further, repeats with no or unknown Blastx hits were considered putatively mobile DNA if they had more than five copies in a single genome. In some cases the classification of a sequence was ambiguous: sequences were then assigned to the general class of Mobile Genetic Element (MGE) based on a compound judgement of their number of copies and nature of similar sequences as determined by Blastn and Blastx.

The above process yielded a genbank file for each of the investigated genomes, where the verified and putative ISs are described with position, species and quality of identification.

### Investigation of local hopping

Test for non-randomness: The probability that at least x IS elements will end up in a certain segment of the genome was computed with the binomial probability density function, which is used to find the probability of at least x successes out of n trials, given the probability p for success. In this case, a “success” occurred if an IS element were within a certain distance from another IS element. The probability p of this occurring is equal to distance divided by replicon size, if the placement of IS elements on the replicon is random. For n IS elements to end up within a certain distance from another IS element, we must get n-1 successes, since there is a 100% chance of one IS element being somewhere on the replicon, but a lower chance that more elements will be located within a certain distance from it. Likewise, the number of trials n is equal to (total number of IS elements-1). In this computation, only IS elements with lengths >70% of the full sequence were counted.

Test if uncorrected p-distance correlates with genomic distance:

- If IS elements jump locally rather than randomly across the genome, closely located IS elements would reasonably have closely related nucleotide sequences, while IS elements separated by larger distances in the genome would have undergone several transposition events and would have deviated from each other due to mutations accumulated over time.
- To test this, all IS elements with lengths >90% of the full sequence were aligned using the built-in Geneious 5.6.6 [37] algorithm for multiple alignments (default parameters). This produced a matrix where the percentage of bases which are identical between two IS elements (the uncorrected p-distance) were given for every pair of IS elements. The uncorrected p-distances for each pair were plotted against the distance between the two IS elements and a linear model was fitted (lm function implemented in R [38]). The p-value generated by this test indicates the probability that the slope of the linear regression is really zero, i.e. that genomic distance has no impact on the uncorrected p-distance between a pair of IS elements. Although the fit was done using a linear curve, it is more likely that the patterns follows a more complex, but still negative, model.
- To exemplify the genomic co-location of similar IS elements, a circular tree was drawn on top of a circle representing the NoAz genome. This tree was constructed by first aligning the NoAz_R_21 elements present in the NoAz genome (same procedure as above). Next the Geneious 5.6.6 Tree Builder was used to construct a neighbour-joining tree (Tamura-Nei distance model, IS element at position 800870 used as out group).

### Pseudogenes in proximity to insertion sequences

A python program was constructed to count the number of pseudogenes within 5,10, 50, 100, 200 and 500 kbp of all annotated genes and IS elements in all contigs of the investigated organisms, using the genbank files produced above together with the original genbank files from NBCIs organism database (NCBI bacterial genome database). For each organism, the count of pseudogenes in the vicinity of non-IS genes was compared to the count of pseudogenes in the vicinity of IS elements, and subjected to the Wilcoxon rank sum test (standard parameters, double-sided) using R [38]. Since the question at hand was whether IS elements affected the integrity of genes native to the organism, the pseudogene count and statistical test were also carried out with IS-like pseudogenes excluded from the count, leaving only “native” genes. Furthermore a count was performed on a dataset where pseudogenes within 1000 bp of the gene or IS element were omitted, since pseudogenes this close to an IS may possibly be unidentified fragments belonging to the IS element nearby. These graphs were almost identical to the first.

## Additional files

Additional file 1:
**Detailed information of repeats found, by genome.** Organism: Name of bacterium. Abbreviation: Abbreviation of bacterium name. GC content: GC content of bacterium genome. Taxonomy ID: NCBI taxonomy ID of organism. Genome size: Size of organism genome, in Mbps. Number of contigs: Number of contigs obtained in in the sequencing of the genome. Genome closed: Have all contigs been found and assigned a position in the genome?. Number of IS hits: Number of found positions with IS elements. Number of phage hits: Number of found positions with phage remains. Number of MGE hits: Number of found positions with putatively mobile DNA. Number of DNA interacting hits: Number of found positions with DNA interacting repeats. Number of unknown but repeated hits: Number of found positions with repeated but unidentified sequences. Number of genes: Number of genes annotated for organism. Number of pseudogenes: Number of pseudogenes annotated for organism. Percentage of pseudogenes: Number of pseudogenes/number of genes. Genome occupied by ISs: Total length of all IS elements, in bp. Fraction of genome occupied by ISs: Total length of IS/Total size of genome. Genome occupied by phages: Total length of all phage remains, in bp. Fraction of genome occupied by phages: Total length of phage remains/Total size of genome. Genome occupied by DNA interacting repeats: Total length of DNA interacting repeats, in bp. Genome occupied by putative MGEs: Total length of putatively mobile repeats, in bp. Genome occupied by unidentified repeats: Total length of highly repeated unidentified sequences, in bp. Size of genome without repeats: Size of genome when all repeats are removed. Size distribution of found sequences: The size distribution of the repeats found in the organism, expressed as a percentage of the original length of the repeats (as decided by RepeatScout or ISfinder). Additional file 2:
**Table of found repeated sequences.** Repeat name: Name of the repeat. Classification: indicator of the kind of repeated sequence, described at bottom of file Length: The length of the repeat as found by RepeatScout, or listed in ISfinder. Family: What IS family the repeat belongs to according to the ISfinder family classification. “-” indicate the repeat is too dissimilar to any known family to be classified into one. “LOW” indicates an expect value above 10e-3 for the blast search used to determine family. ISfinder blastx result: The best result when sequence was used as query in a blastx search against the aa sequences at the ISfinder site. ISfinder score fraction: Score from the above search, divided with the length of the query sequence. NCBI blastx result: The best result when sequence was used as query in a blastx search against the nr sequences at NCBI. NCBI blastx score fraction: Score from the above search, divided with the length of the query sequence. Size distribution of found sequences (% of total length): The size distribution of the repeats found in all organisms, expressed as a percentage of the original length of the repeat (as decided by RepeatScout or ISfinder). Sequence: The original sequence of the repeats, as decided by RepeatScout or ISfinder. Additional file 3:
**Detailed table of repeats found.** Classification data: reason for assigning the repeat a certain classification. Length: Length of the repeat. Total number of hits: Total number of this repeat, in all lengths, in all organisms. Size distribution of found sequences (% of total length): Size distribution of the repeats found in all organisms. Found in species: All species of bacteria where at least one copy of the repeat was observed. ISfinder blastx hit: Best score blastx hit against a database of ISfinder amino acid sequences. Local blastn hit on repeats and ISfinder sequences: Best score blastn hit against a database of ISfinder nucleotide sequences and RepeatScout generated repeats. ISfinder blastn hit: Best score blastn hit against a database of ISfinder nucleotide sequences only. NCBI nr blastx best score hit: Best score blastx hit against NCBI’s nr database. Best score NCBI nr blastx known protein: Best score blastx hit against NCBI’s nr database that is not “unknown” or “hypothetical”. Best NCBI blastx “transposase” hit: Best score blastx hit against NCBI’s nr database that indicates sequence is transposase. Best NCBI blastx phage hit: Best score blastx hit against NCBI’s nr database that indicates sequence is of phage origin. Best NCBI blastx phage hit: Best score blastx hit against NCBI’s nr database that indicates sequence is DNA interacting. Best NCBI blastx putative MGE hit: Best score blastx hit against NCBI’s nr database indicating mobility or self-replication. Known non-MGE hit with NCBI blastx: Best score blastx hit against NCBI’s nr database that indicates that the sequence encodes a non-MGE protein. Best score NCBI blastn nt hit: Best score blastn hit against NCBI’s nt database. Sequence: Nucleotide sequence of the repeat. Additional file 4:
**Plots of uncorrected p-distance compared to genomic separation for some repeats in NoAz.** Plots of decreasing uncorrected p-distance as sequences move further from each other on the chromosome. Each point in the graphs represents a pair of IS elements of the same type. Their location on the X-axis represents the number of nucleotides separating the pair, and the location of the Y-axis represents uncorrected p-distance, i.e. the fraction of nucleotides that are identical in the pair. The red line indicates most likely slope for the decrease of uncorrected p-distance with genomic distance. “Slope” indicates the lines equation, and “p” the likelihood that the slope of the line is actually zero. Since so many factors (varying “jump” distance, genomic rearrangements, mutations) influence IS placement and similarity, it is not surprising that the spread around the line is very large. Additional file 5:
**Graphs of decrease in pseudogene enrichment with increasing distance from IS elements for all investigated organisms.** X-axis: Distance from gene or IS elements within which all pseudogenes are counted. Y axis: Pseudogene enrichment, i.e. (average number of pseudogenes within distance to IS elements)/(average number of pseudogenes within distance to regular genes). Green circles indicate that the difference in pseudogene enrichment is statistically significant (p < 0.05), red circles indicate that the difference is not significant.

## Abbreviations

Bp: Base pair
MGE: Mobile Genetic Element
IS: Insertion Sequence
NoAz: ‘*Nostoc azollae*’ 0708
nt: Nucleotide

## References

[1] Mahillon J, Chandler M (1998). Insertion sequences. Microbiol Mol Biol Rev.

[2] Siguier P, Gourbeyre E, Chandler M (2014). Bacterial insertion sequences: their genomic impact and diversity. FEMS Microbiol Rev.

[3] Skipper KA, Andersen PR, Sharma N, Mikkelsen JG: DNA transposon-based gene vehicles - scenes from an evolutionary drive. *J Biomed Sci* 2013, 20. 10.1186/1423-0127-20-92 PMC3878927 24320156

[4] Moran NA, Plague GR (2004). Genomic changes following host restriction in bacteria. Curr Opin Genet Dev.

[5] McCutcheon JP, Moran NA (2012). Extreme genome reduction in symbiotic bacteria. Nat Rev Micro.

[6] Bickhart D, Gogarten J, Lapierre P, Tisa L, Normand P, Benson D (2009). Insertion sequence content reflects genome plasticity in strains of the root nodule actinobacterium Frankia. BMC Genomics.

[7] Pawlowski K, Bisseling T (1996). Rhizobial and actinorhizal symbioses: what are the shared features?. Plant Cell.

[8] Wei J, Goldberg MB, Burland V, Venkatesan MM, Deng W, Fournier G (2003). Complete genome sequence and comparative genomics of Shigella flexneri serotype 2a strain 2457 T. Infect Immun.

[9] Dasilva C, Hadji H, Ozouf-Costaz C, Nicaud S, Jaillon O, Weissenbach J (2002). Remarkable compartmentalization of transposable elements and pseudogenes in the heterochromatin of the Tetraodon nigroviridis genome. Proc Natl Acad Sci.

[10] Walker A, Langridge G (2008). Does my genome look big in this?. Nat Rev Microbiol.

[11] Moore AW (1969). Azolla: Biology and agronomic significance. Bot Rev.

[12] Larsson J, Nylander JA, Bergman B (2011). Genome fluctuations in cyanobacteria reflect evolutionary, developmental and adaptive traits. BMC Evol Biol.

[13] Zheng W, Bergman B, Chen B, Zheng S, Xiang G, Rasmussen U (2009). Cellular responses in the cyanobacterial symbiont during its vertical transfer between plant generations in the Azolla microphylla symbiosis. New Phytol.

[14] Ran L, Larsson J, Vigil-Stenman T, Nylander JAA, Ininbergs K, Zheng W-W (2010). Genome Erosion in a Nitrogen-Fixing Vertically Transmitted Endosymbiotic Multicellular Cyanobacterium. PLoS One.

[15] Tang LF, Watanabe I, Liu CC (1990). Limited multiplication of symbiotic cyanobacteria of Azolla spp. on artificial media. Appl Environ Microbiol.

[16] Deusch O, Landan G, Roettger M, Gruenheit N, Kowallik KV, Allen JF (2008). Genes of Cyanobacterial Origin in Plant Nuclear Genomes Point to a Heterocyst-Forming Plastid Ancestor. Mol Biol Evol.

[17] Reyes-Prieto A, Weber AP, Bhattacharya D (2007). The origin and establishment of the plastid in algae and plants. Annu Rev Genet.

[18] Moreira D, Le Guyader H, Philippe H (2000). The origin of red algae and the evolution of chloroplasts. Nature.

[19] de Alda JAG O, Esteban R, Diago ML, Houmard J (2014). The plastid ancestor originated among one of the major cyanobacterial lineages. Nat Commun.

[20] Cho N-H, Kim H-R, Lee J-H, Kim S-Y, Kim J, Cha S (2007). The Orientia tsutsugamushi genome reveals massive proliferation of conjugative type IV secretion system and host–cell interaction genes. Proc Natl Acad Sci.

[21] Schmitz-Esser S, Tischler P, Arnold R, Montanaro J, Wagner M, Rattei T (2010). The genome of the amoeba symbiont “Candidatus Amoebophilus asiaticus” reveals common mechanisms for host cell interaction among amoeba-associated bacteria. J Bacteriol.

[22] Hise AG, Gillette Ferguson I, Pearlman E (2004). The role of endosymbiotic Wolbachia bacteria in filarial disease. Cell Microbiol.

[23] Parkhill J, Wren BW, Thomson NR, Titball RW, Holden MTG, Prentice MB (2001). Genome sequence of Yersinia pestis, the causative agent of plague. Nature.

[24] Normand P, Lapierre P, Tisa LS, Gogarten JP, Alloisio N, Bagnarol E (2007). Genome characteristics of facultatively symbiotic Frankia sp. strains reflect host range and host plant biogeography. Genome Res.

[25] Parkhill J, Sebaihia M, Preston A, Murphy LD, Thomson N, Harris DE (2003). Comparative analysis of the genome sequences of Bordetella pertussis, Bordetella parapertussis and Bordetella bronchiseptica. Nat Genet.

[26] Thiaucourt F, Manso-Silvan L, Salah W, Barbe V, Vacherie B, Jacob D (2011). Mycoplasma mycoides, from “mycoides Small Colony” to “capri”. A microevolutionary perspective. BMC Genomics.

[27] Oshima K, Kakizawa S, Nishigawa H, Jung H-Y, Wei W, Suzuki S (2003). Reductive evolution suggested from the complete genome sequence of a plant-pathogenic phytoplasma. Nat Genet.

[28] Siguier P, Pérochon J, Lestrade L, Mahillon J, Chandler M (2006). ISfinder: the reference centre for bacterial insertion sequences. Nucleic Acids Res.

[29] Pade N, Compaoré J, Klähn S, Stal LJ, Hagemann M (2012). The marine cyanobacterium Crocosphaera watsonii WH8501 synthesizes the compatible solute trehalose by a laterally acquired OtsAB fusion protein. Environ Microbiol.

[30] Thompson AW, Foster RA, Krupke A, Carter BJ, Musat N, Vaulot D (2012). Unicellular cyanobacterium symbiotic with a single-celled eukaryotic alga. Science.

[31] Alam J, Vrba JM, Cai Y, Martin JA, Weislo LJ, Curtis SE (1991). Characterization of the IS895 family of insertion sequences from the cyanobacterium Anabaena sp. strain PCC 7120. J Bacteriol.

[32] Cai Y (1991). Characterization of insertion sequence IS892 and related elements from the cyanobacterium Anabaena sp. strain PCC 7120. J Bacteriol.

[33] Boyd EF, Hartl DL (1997). Nonrandom location of IS1 elements in the genomes of natural isolates of Escherichia coli. Mol Biol Evol.

[34] Newton IG, Bordenstein S (2011). Correlations Between Bacterial Ecology and Mobile DNA. Curr Microbiol.

[35] Si-Ping Z, Bin C, Xiong G, Wei-Wen Z (2008). Diversity analysis of endophytic bacteria within Azolla microphylla using PCR-DGGE and electron microscopy. Chin J Agric Biotechnol.

[36] Price AL, Jones NC, Pevzner PA (2005). De novo identification of repeat families in large genomes. Bioinformatics.

[37] Kearse M, Moir R, Wilson A, Stones-Havas S, Cheung M, Sturrock S (2012). Geneious Basic: an integrated and extendable desktop software platform for the organization and analysis of sequence data. Bioinforma Oxf Engl.

[38] R Core Team (2014). R: A Language and Environment for Statistical Computing.

