# Supplementary material for: Local hopping mobile DNA implicated in pseudogene formation and reductive evolution in an obligate cyanobacteria-plant symbiosis
Source: BMC Genomics. 2015 Mar 17;16(1):193. doi: 10.1186/s12864-015-1386-7 (PMC4369082; doi:10.1186/s12864-015-1386-7)

Repeat alignment identities versus distance on chromosome for some repeats in NoAZ

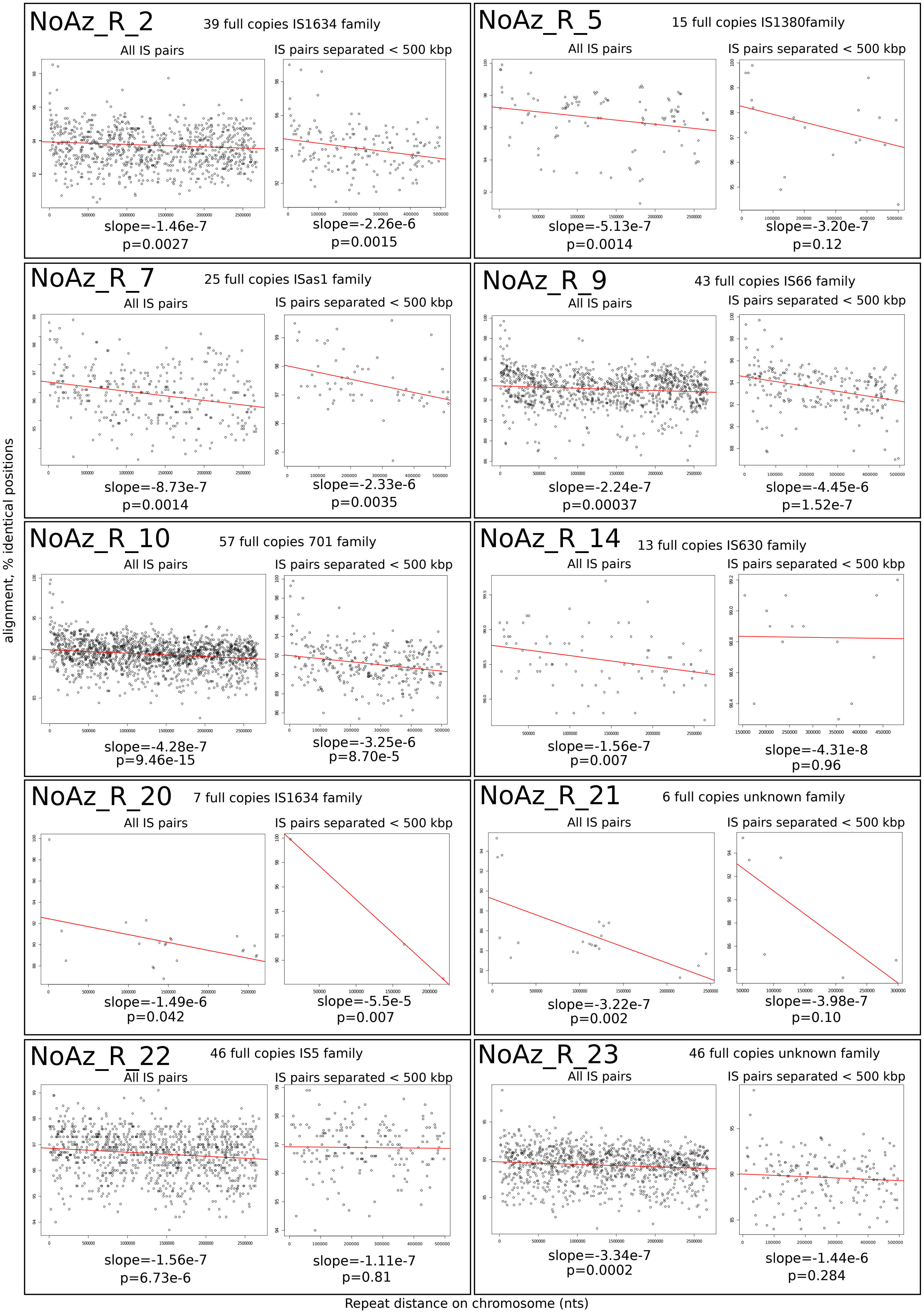

Supplement: Additional file 4: — Plots of uncorrected p-distance compared to genomic separation for some repeats in NoAz. Plots of decreasing uncorrected p-distance as sequences move further from each other on the chromosome. Each point in the graphs represents a pair of IS elements of the same type. Their location on the X-axis represents the number of nucleotides separating the pair, and the location of the Y-axis represents uncorrected p-distance, i.e. the fraction of nucleotides that are identical in the pair. The red line indicates most likely slope for the decrease of uncorrected p-distance with genomic distance. “Slope” indicates the lines equation, and “p” the likelihood that the slope of the line is actually zero. Since so many factors (varying “jump” distance, genomic rearrangements, mutations) influence IS placement and similarity, it is not surprising that the spread around the line is very large. [file 12864_2015_1386_MOESM4_ESM.pdf]
