# Supplementary material for: Local hopping mobile DNA implicated in pseudogene formation and reductive evolution in an obligate cyanobacteria-plant symbiosis
Source: BMC Genomics. 2015 Mar 17;16(1):193. doi: 10.1186/s12864-015-1386-7 (PMC4369082; doi:10.1186/s12864-015-1386-7)
Supplement: Additional file 5: — Graphs of decrease in pseudogene enrichment with increasing distance from IS elements for all investigated organisms. X-axis: Distance from gene or IS elements within which all pseudogenes are counted. Y axis: Pseudogene enrichment, i.e. (average number of pseudogenes within distance to IS elements)/(average number of pseudogenes within distance to regular genes). Green circles indicate that the difference in pseudogene enrichment is statistically significant (p < 0.05), red circles indicate that the difference is not significant. [file 12864_2015_1386_MOESM5_ESM.zip › Additional_files_5/Cya7425graph.pdf]

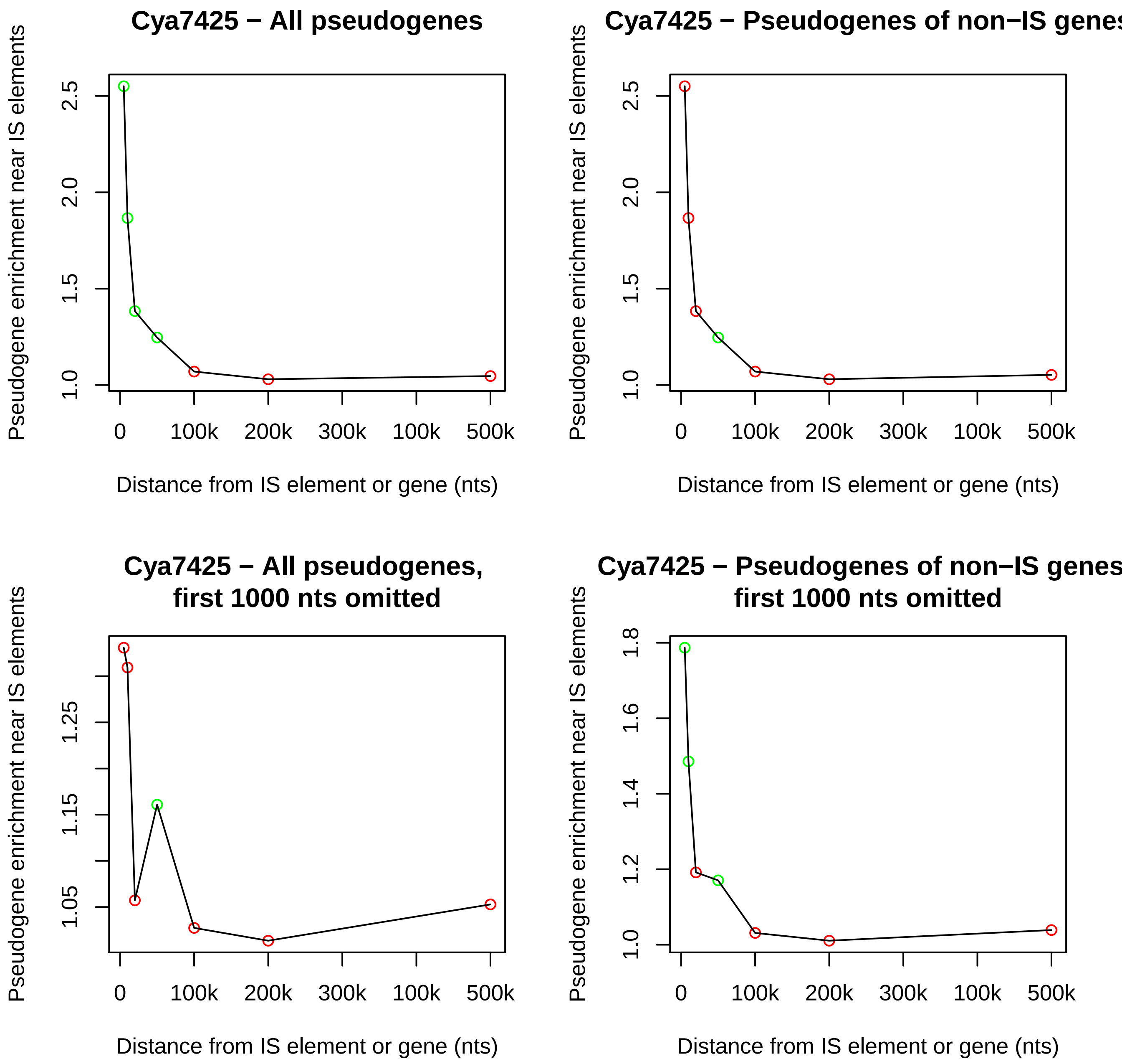
Cya7425 − All pseudogenes
Cya7425 − Pseudogenes of non−IS genes
Cya7425 − All pseudogenes, first 1000 nts omitted
Cya7425 − Pseudogenes of non−IS genes first 1000 nts omitted
Pseudogene enrichment near IS elements
Distance from IS element or gene (nts)
1.0
1.5
2.0
2.5
1.05
1.15
1.25
1.2
1.4
1.6
1.8
0
100k
200k
300k
100k
500k
